# CRISPR-cas9 screening identified lethal genes enriched in Hippo kinase pathway and of predictive significance in primary low-grade glioma

**DOI:** 10.1186/s10020-023-00652-3

**Published:** 2023-05-14

**Authors:** Maimaitili Mijiti, Aierpati Maimaiti, Xiaoqing Chen, Maidina Tuersun, Miershayiti Dilixiati, Yilidanna Dilixiati, Guohua Zhu, Hao Wu, Yandong Li, Mirzat Turhon, Aimitaji Abulaiti, Nuerailijiang Maimaitiaili, Nadire Yiming, Maimaitijiang Kasimu, Yongxin Wang

**Affiliations:** 1 Department of Neurosurgery, Neurosurgery Centre, The First Affiliated Hospital of Xinjiang Medical University, Xinjiang 830054 Urumqi, China; 2 The First Affiliated Hospital of Xinjiang Medical University, Urumqi, 830054 Xinjiang China; 3 Xinjiang Medical University, Urumqi, 830011 Xinjiang China; 4 Department of Neurointerventional Surgery, Beijing Neurosurgical Institute, Capital Medical University, Beijing, 100070 China; 5 Department of Neurointerventional Surgery, Beijing Tiantan Hospital, Capital Medical University, Beijing, 100070 China

**Keywords:** CRISPR-cas9 screening, Low-grade glioma, Hippo signaling pathway, Prognosis, Signature, WGCNA

## Abstract

**Background:**

Low-grade gliomas (LGG) are a type of brain tumor that can be lethal, and it is essential to identify genes that are correlated with patient prognosis. In this study, we aimed to use CRISPR-cas9 screening data to identify key signaling pathways and develop a genetic signature associated with high-risk, low-grade glioma patients.

**Methods:**

The study used CRISPR-cas9 screening data to identify essential genes correlated with cell survival in LGG. We used RNA-seq data to identify differentially expressed genes (DEGs) related to cell viability. Moreover, we used the least absolute shrinkage and selection operator (LASSO) method to construct a genetic signature for predicting overall survival in patients. We performed enrichment analysis to identify pathways mediated by DEGs, overlapping genes, and genes shared in the Weighted correlation network analysis (WGCNA). Finally, the study used western blot, qRT-PCR, and IHC to detect the expression of hub genes from signature in clinical samples.

**Results:**

The study identified 145 overexpressed oncogenes in low-grade gliomas using the TCGA database. These genes were intersected with lethal genes identified in the CRISPR-cas9 screening data from Depmap database, which are enriched in Hippo pathways. A total of 19 genes were used to construct a genetic signature, and the Hippo signaling pathway was found to be the predominantly enriched pathway. The signature effectively distinguished between low- and high-risk patients, with high-risk patients showing a shorter overall survival duration. Differences in hub gene expression were found in different clinical samples, with the protein and mRNA expression of REP65 being significantly up-regulated in tumor cells. The study suggests that the Hippo signaling pathway may be a critical regulator of viability and tumor proliferation and therefore is an innovative new target for treating cancerous brain tumors, including low-grade gliomas.

**Conclusion:**

Our study identified a novel genetic signature associated with high-risk, LGG patients. We found that the Hippo signaling pathway was significantly enriched in this signature, indicating that it may be a critical regulator of tumor viability and proliferation in LGG. Targeting the Hippo pathway could be an innovative new strategy for treating LGG.

**Supplementary Information:**

The online version contains supplementary material available at 10.1186/s10020-023-00652-3.

## Introduction

Low-grade gliomas (LGG) have been demonstrated as being among the most prevalent primary tumors affecting the central nervous system and consist of WHO grade II and III gliomas (Louis et al. 2007; Li et al. 2021). Although molecular features including isotope dehydrogenase 1 and 2 genes (IDH1/IDH2), PTEN, EGFR, ATRX, TPP53, coding status of chromosome arms 19q and 1p, Chr 7 gain/Chr 10 loss, Chr19/20 co-gain, have significantly distinguished different classes of LGG (Chiang et al. 2020). LGG has significant heterogeneity that hinders improved patient outcomes (Wang et al. 2021). Until now, several approaches have been used to treat gliomas, such as lytic virus therapy, targeted therapy, immunotherapy, chemotherapy, radiation therapy, and surgery, but clinical outcomes for LGG patients have not significantly improved (Cai et al. 2020). Therefore, it is essential to improve the efficacy of treatment for patients with LGG. In addition to conventional therapies, there is growing interest in the emerging CRISPR/Cas9 gene-editing system.

CRISPR/Cas9 system is widely found in prokaryotic genomes and is an acquired immune defense mechanism that has evolved in bacteria and archaea in response to viral and plasmid invasion (Usman et al. 2020; Louradour et al. 2019). The CRISPR/Cas9 system mainly consists of the Cas9 protein and single-stranded guide RNA (sgRNA) (Peng et al. 2018). Cas9 protein recognizes a specific DNA sequence under the guidance of sgRNA. The Cas9 protein can cut the different target sites through the principle of base complementary pairing to achieve the double-strand break of DNA (Moses et al. 2019; Yang et al. 2020). In addition CRISPR/Cas9 can also be used for gene expression regulation (transcriptional activation/repression), epigenetic modifications, and genomic imaging (Amjad et al. 2020; Khanzadi and Khan 2020). This application relies on the resolution of the Cas9 protein structure (Gangopadhyay et al. 2019). Cas9, a multifunctional protein, possesses two nuclease structural domains, HNH and RuvC. The HNH domain cuts the DNA strand complementarily paired with crRNA, and the RuvC domain cuts the other strand of double-stranded DNA (Stovicek et al. 2017; Wu et al. 2020). Cas9 becomes a single-stranded cleaved protein if one of the two structural domains is mutated, and if both are mutated, Cas9 becomes a protein with only DNA-binding activity (Young et al. 2019). dCas9 stands for “dead” or “catalytically inactive” Cas9, which is a modified form of the Cas9 protein commonly used in CRISPR gene editing. Unlike the active Cas9 protein, which can cut DNA, dCas9 cannot cleave DNA, but it retains its ability to specifically bind to a target DNA sequence. This makes it useful for gene regulation, as it can be directed to bind to a specific gene sequence and either block or activate its expression without permanently altering the DNA sequence. When dCas9 binds to the coding region or promoter region of a gene, it affects the activity of RNA polymerase and thus transcription, a method also known as CRISPRi (Mahas et al. 2018).In humans and yeast, if the dCas9 protein is expressed in fusion with VP64 or KRAB, it brings about transcriptional activation and transcriptional repression, respectively (Kleinjan et al. 2017; Wen et al. 2016).

Genome-wide knockdown technologies developed based on the CRISPR/Cas9 system are making their mark in numerous areas of oncology research. These technologies contribute to the comprehension of the impact of knocking out established genes on biological phenotypes on a genome-wide scale. Recently, it was shown that CRISPR-Cas9-mediated knockdown of the TIM3 gene in human natural killer cells enhanced the growth inhibition of human glioma cells (Morimoto et al. 2021). In addition, it was discovered that the CRISPR/Cas9 system specifically targets EGFR exon 17, resulting in the inhibition of the activation of NF-kB by epigenetically modulating UBXN1 in EGFRwt/vIII glioma cells. Thus, this mechanism indicates that CRISPR/Cas9 is a viable treatment modality for GBM patients with EGFR mutations and EGFR amplification (Huang et al. 2017). Moreover, it was also found in mice with glioblastoma and injected with CRISPR-LNP targeting PLK1, an enzyme essential for cell division, which successfully caused apoptosis in tumor cells after editing the gene encoding this enzyme within the tumor cells. Therefore, the results showed that mice with a single intracerebral injection of CRISPR-LNP compared to the control group had a gene-editing efficiency of 70%. In addition, the median survival of the mice increased from 32.5 days to more than 48 days, and 30% of the mice survived for at least 60 days, while the control mice at 40 days were all dead (Rosenblum et al. 2020). Compared with pooled guide RNA libraries, CRISPR/cas9 may be used in a high-throughput method to filter for genes associated with specific biochemical phenotypes or illnesses (Esvelt et al. 2014). This “*phenotype to genotype*” strategy plays a role in modifying gene expression by choosing cells that exhibit the phenotype of interest (Pereira and Weinshilboum 2009), followed by sequence analysis of the desired perturbations, which may identify genes that are involved in cell viability. In parallel, to determine the influence of single-gene knockouts on cell survival, a large-scale cancer-dependent loss-of-function screen was carried out in several well-characterized cancer cell lines. The Cancer Dependency Map (DepMap) website provides these data sets. This approach allows for the identification of key genes and regulatory pathways involved in a particular phenotype, which can then be manipulated to alter gene expression and cellular behavior. By emphasizing this strategy in our study, we were able to demonstrate the potential of using dCas9-based epigenetic editing to modify the expression of genes involved in cell differentiation and reprogramming. This approach has significant implications for both basic research and clinical applications, and underscores the importance of understanding the relationship between genotype and phenotype in the context of complex biological systems.

The Hippo pathway is a significantly conserved cascade signaling pathway in drosophila and mammals that plays a role in diverse biological activities, such as cell viability, proliferation, apoptosis, differentiation, cell fate determination, and tissue and organ size and homeostasis through the regulation of key target genes (Seo et al. 2020; Clattenburg et al. 2015). The abnormal signaling of the Hippo pathway is also implicated in multiple pathologies, including cancer and immunity-related diseases (Merrick et al. 2019). YAP/TAZ is the predominant transcription co-activator downstream of the Hippo pathway, shuttling between the cytoplasm and the nucleus (Shuttle) (Kim et al. 2020). The YAP/TAZ protein is the hub of the Hippo pathway, where a variety of upstream signaling molecules act directly or indirectly on YAP/TAZ, mainly to regulate the localization of YAP/TAZ, i.e., to regulate YAP/TAZ retention in the cytoplasm or nuclear localization (localization in the nucleus and interaction with corresponding transcriptional factors to modulate the target genes expression) (Corre et al. 2019). According to a previous study, NF2-deficient PRCC cancers that have lost the capacity of regulating the Hippo signaling pathway could be treated with dasatinib, which targets Yes in YAP-activated tumors and inhibits its expression (Sourbier et al. 2018). Another study showed that TFAP2C enhances CSCs’ properties and chemoresistance through transcriptional activation of ROCK1 and ROCK2, which are negative regulators of Hippo signaling, leading to deactivation of Hippo signaling in colorectal cancer cells (Wang et al. 2018). Moreover, the YAP/TAZ-TEAD transcriptional factor complex is an application target for oncogenic transformation. The YAP locus is shown to be upregulated at different frequencies in human and mouse tumors, such as medulloblastoma, lung, pancreatic, esophageal, hepatocellular, and breast cancers (Pan 2010). In LGG, LATS2 suppresses the proliferative and metastatic ability of cells via the Hippo signaling pathway (Guo et al. 2019). These studies suggest that the Hippo signaling pathway exerts a critical role in treating cancer.

The biological mechanisms involved in cell viability are complicated. Nonetheless, the cellular vulnerability of LGG has not been investigated in-depth in a systematic manner. Furthermore, the pathways via which these genes function and their prognostic value, have never been fully investigated. Thus, the field of bioinformatics plays a crucial role in identifying risk signatures, which can provide valuable insights into disease progression and potential treatment options. In this study, we utilized various bioinformatics tools to identify genes associated with the Hippo signaling pathway and their potential impact on cancer risk. Through a combination of computational analysis and experimental validation, we were able to identify three genes, SOX9, RPE65, and LSM2, as potential biomarkers for cancer risk assessment. Our findings highlight the importance of bioinformatics in identifying and validating risk signatures for disease diagnosis and treatment.

## Materials and methods

### Identification of gene-knockout effect based on CRISPR-Cas9 and CERES

**Fig. 1:**
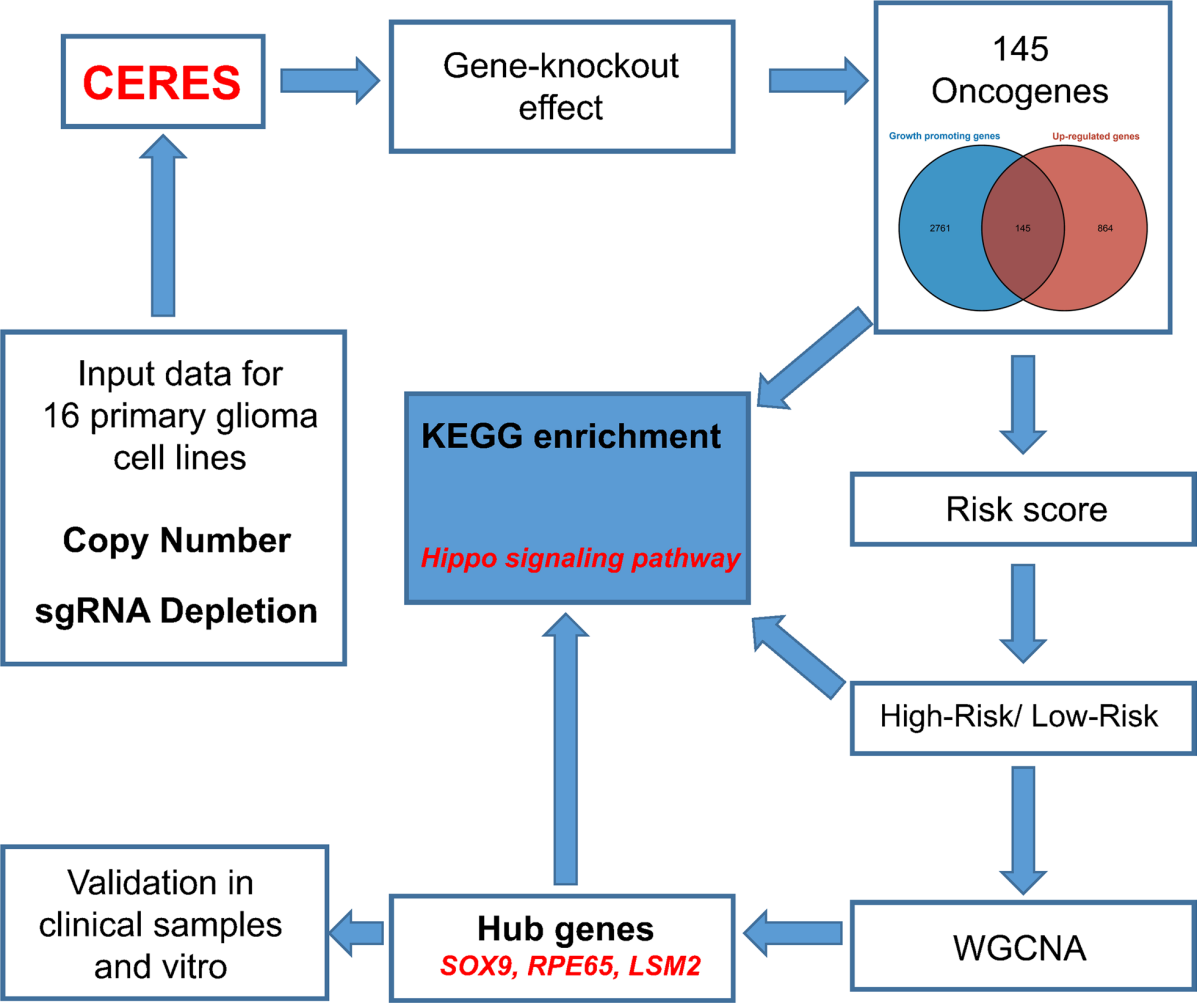
Flow chart of our study

CERES, a computational method to estimate gene dependency levels from CRISPR-Cas9 essentiality screens while accounting for the copy-number-specific effect (Meyers et al. 2017). Results of running CERES on 16 primary non-metastatic glioma cell lines screened with the Avana sgRNA library, namely CAS1, DKMG, GB1, HS683, KALS1, KNS60, KNS81, LN18, M059K, SF295, SNU201, T98G, U118MG, U251MG U87MG, and YKG1, were download from Depmap database. CERES R package (version 1.0.0) was used for the purpose of calculating the dependency scores, aiming to detect genes that are critical for the ability of the cells lines to survive. Notably, a negative value for a gene score implies that silencing it suppresses the survival condition of a cell line, whereas a positive value demonstrates that silencing it enhances proliferation and survival. The growth inhibitor and growth promotor genes were defined using thresholds of 0.5 and − 0.5, respectively. DEGs between tumor and normal samples were calculated using the “limma” R package (version 3.50.3). Adjusted *p-value* < 0.05 and absolute fold changes > 1 were used as thresholds for selecting DEGs. Growth inhibitor genes were intersected with down-regulated genes, whereas growth promoter genes were intersected with up-regulated genes. The flow chart of our study was shown in Fig. 1.

### Datasets

RNA-Seq data of 491 LGG samples from the TCGA database (Level-3 HTseq-FPKM) and 103 normal cortical samples from the GTEx project were included in this study. These were used as normal sample controls, and after excluding non-coding RNA, differential analysis was performed. In addition, 10 samples with duplicate sequencing, no survival status, overall survival (OS) time < 1 day, no clear WHO classification, and non-primary LGG were excluded for subsequent modeling. LGG is defined as diffuse low-grade and intermediate-grade glioma (WHO grades II and III). Datasets were defined using International Classification of Diseases for Oncology, third edition (ICD-O-3) codes 9382/3, 9400/3, 9401/3, 9450/3, and 9451/3 (Gittleman et al. 2020; Liu et al. 2018). Finally, 481 LGG patients were enrolled in the study.

### Processing of data in low-grade glioma

The Cancer Genome Atlas (TCGA) database is a collection of genomic data from different types of cancer, including gliomas. On the other hand, the Chinese Glioma Genome Atlas (CGGA) database is a large-scale genomic data resource that is specifically focused on gliomas, and it contains a wealth of genomic information such as gene expression profiles, DNA methylation patterns, and copy number variations. The 481 samples from the TCGA-LGG database were randomly split 7:3 using the caret package, with TCGA-LGG-1 as the modeling cohort. TCGA-LGG-2 was used as the internal validation cohort. In brief, the remaining 481 samples were randomized into two groups, with 337 samples in LGG-1 cohort and 144 samples in LGG-2 cohort. Meanwhile, the GSE16011 dataset based on the GPL570 platform was extracted from the GEO database, and 80 of the LGG patients with WHO classification II–III were retained as the external validation group (GEO-LGG). The GPL570 platform, also known as the Affymetrix Human Genome U133 Plus 2.0 Array, contains probes for over 47,000 transcripts, making it a commonly used tool for gene expression studies. The CGGA database was used to download the CGGA-693 project (CGGA-LGG-1, 332 patients), and the CGGA-325 project (CGGA-LGG-2, 162 patients) containing complete survival information of LGG patients as the external validation group. In summary, the TCGA-LGG-1 cohort was used for modeling, the TCGA-LGG-2 cohort for internal validation, and the GEO-LGG, CGGA-LGG-1, and the CGGA-LGG-2 cohorts for external validation. In addition, the “sva” R package (version 3.42.0) was used to perform normalization for the genes involved in the modeling, thus ensuring comparability of validation.

### LASSO regression analysis

In the TCGA-LGG cohort, the patients were classified into two datasets at random with a 7:3 ratio, namely the internal training and internal validation datasets. Risk models were constructed with the aid of the LASSO model, aiming to eliminate highly correlated genes. By incorporating gene expression values that their LASSO-Cox coefficients have weighted, risk score formulas were developed. The prognostic significance of the risk scores was evaluated by performing univariate and multivariate Cox regression analyses using the entire dataset as well as the external validation dataset. Time-dependent subject operating characteristic curves (ROC) were used to evaluate the prediction performance of risk scores with conventional clinical and pathological characteristics (Zhang et al. 2022). For the purpose of plotting ROC curves and calculating the area under the curve (AUC), the “pROC” R package (version 1.18.0) was utilized.

### Enrichment analysis

Gene Ontology (GO) is a classification system that is used for annotating cell components, molecular functions, and biological processes. Gene pathways were annotated using the Kyoto Encyclopedia of Genes and Genomes (KEGG). The “clusterProfiler” R package (version 4.2.2) was employed to conduct the GO and KEGG analyses. The *q-value* < 0.05 and *p-value* < 0.05 were established as the criteria for identifying significantly enriched pathways. We performed GO and KEGG analyses to gain insights into the potential biological functions and signaling pathways that may be affected by the genes identified in our differential expression analysis. This analysis allows us to infer the potential roles of these genes in the biological processes, molecular functions, and cellular components of the glioma development and progression.

### Creation of the PPI network and detection of hub

The STRING website provides information about available and anticipated protein–protein interactions, aiding the creation of PPI networks. Subsequently, the overlapping gene interactions were visualized with the aid of the Cytoscape software (version 3.8.0).

### Weighted correlation network analysis

In the WGCNA R package (version 1.71), all DEGs satisfying the *p-value* < 0.05 in normal and tumor samples in the CGGA-LGG-1 cohort were used as input. Each sample was clustered well, with only a single outlier sample excluded using a shear line of 120 as the threshold. Subsequently, a soft threshold from 1 to 20 was used for topology calculation (Feng et al. 2022), and the optimal soft threshold was determined to be 10. After converting the correlation matrix into an adjacency matrix according to the soft threshold, it was then converted into a topological overlap matrix (TOM) for mean linkage hierarchical clustering. Furthermore, the associated modules were subjected to classification based on the TOM, with the number of genes in each module not below 50. In the present research, the shear height of the gene modules was 0.2 (Cheng et al. 2022), and similar modules were integrated. Finally, the correlation between the merged modules and the high-risk population was obtained with the help of the Pearson method.

### Clinical samples

We chose tissues from patients with intractable epilepsy as the “normal” control tissues for our study because these samples were obtained from patients who did not have brain tumors or other neurological disorders. While these tissues may not be completely “normal” in the traditional sense, they represent a suitable control group for our study given the absence of tumors or other neurological disorders. In our study, we collected the 10 LGG tissues and 10 normal brain tissues were prepared for extracting RNA and protein. In addition, we obtained 10 tumor tissues from LGG patients and 10 brain tissues from patients with intractable epilepsy for IHC sections. The study of human samples was approved by the Medical Ethical Committee of the first affiliated hospital of Xin-Jiang medical university.

### Immunohistochemistry

The detailed protocol about western blot, qRT-PCR and IHC were displayed in previous references (Zhu et al. 2015; Feng et al. 2020). In brief, paraffin-embedded tissue sections were baked, dehydrated, and subjected to antigen repair on tissue slides. Then, endogenous peroxidase was inactivated with 3% hydrogen peroxide for 15 min. The non-specific antigen was blocked using 5% BSA for 30 min at room temperature and incubated with primary antibody overnight at 4 °C. The next day, slides are incubated with the cognate secondary antibody for 1 h at room temperature. Then, DAB staining, hematoxylin staining, followed by dehydration in gradient ethanol and clear treatment of slides in xylene.

### Western blot

Proteins from different tissues were extracted with RIPA buffer, then separated on SDS/PAGE and transferred to PVDF membranes, incubated overnight with the corresponding primary antibodies and incubated with HRP-conjugated secondary antibodies, and finally the luminescence signal was detected by ECL. The following antibodies were used: GAPDH (1:2000, GB15002, Servicebio), SOX9 (1:1000, 67439-1-IG, proteintech), RPE65 (1:1000, 17939-1-AP, proteintech), LSM2 (1:1000, 46289, SAB). GAPDH was used for normalization. ImageJ software was used to evaluate and quantify the gray value for 20 samples.

### qRT-PCR

Total RNA was extracted from the tissues using TRIzol and then converted to cDNA using Servicebio®RT First Strand cDNA Synthesis Kit. Real-time PCR was performed using SYBR Green qPCR Master Mix (None ROX). Relative mRNA expression was determined based on CT values and normalized by the GAPDH expression levels. The cycling conditions used for PCR amplification were as follows: an initial denaturation step at 95 °C for 5 min, followed by 30 cycles of denaturation at 95 °C for 30 s, annealing at 55 °C for 30 s, and extension at 72 °C for 30 s, and a final extension step at 72 °C for 5 min. Sequences of primers were as follows:

- GAPDH forward: 5′-GGAAGCTTGTCATCAATGGAAATC-3′, GAPDH reverse: 5′-TGATGACCCTTTTGGCTCCC-3′;
- SOX9 forward: 5′-GTCAACGGCTCCAGCAAGAA-3′, SOX9 reverse: 5′-CGTTCTTCACCGACTTCCTCC-3′;
- RPE65 forward: 5′-TGGGCCAGGACTCTTTGAAG-3′, RPE65 reverse: 5′-TGCGGATGAACCTTCTGTGG-3′;
- LSM2 forward: 5′-TCGTGGAACTAAAGAATGACCTGA-3′, LSM2 reverse: 5′-CATCCTGTAGCAACTGTGTGTCG-3′.

### Cell culture

The cell lines were purchased from iCell Bioscience Inc (Shanghai, China). The human astrocyte cell line NHA and glioma cell lines (U87, U251, and T98G) were employed in our study, because there are no particular LGG cell lines available. The Dulbecco’s modified Eagle’s medium (10% fetal bovine serum) was used to cultivate the cells.

### Cell counting Kit-8 assay

U87 and T98G cells was assessed with the Cell Counting Kit-8 (Dojindo Molecular Technologies, Kyushu, Japan) reagent according to the manufacturer’s instructions (Zhou et al. 2022). Briefly, cells were seeded in 96-well plates and treated with different concentrations of compounds for 24, 48 or 72 h. After treatment, CCK-8 reagent was added to each well, and the plates were incubated for an additional 1.5 h. The absorbance was measured at 450 nm using a microplate reader (Synergy HTX, BioTek Instruments, Inc., Winooski, VT, USA).

### Invasion assays

2 × 10^4^ cells were added into Matrigel-coated upper Transwell chambers for the invasion assay. The lower chambers were filled with DMEM containing 10% fetal bovine serum. After incubation at 37 °C for 24 h, cells on the lower surface of the membrane were fixed and stained. The pore size of the Transwell filter was 8 μm, and the filters were stained with crystal violet.

### Lentivirus infection assay

Short hairpin RNA against RPE65 (sh-RPE65) and a negative control shRNA (sh-NC) were designed by genepharma (Shanghai, China). The lentivirus pLent-sh RPE65-GFP-Puro or its negative control (NC) pLent-GFP-Puro was used to infect U87 and T98G cells. 2 g/mL puromycin was added 2 days after the cells were infected with lentivirus.

### Wound healing

U87 and T98G cells were seeded in a 6-well plate and allowed to grow to confluence. A straight scratch was made in the center of each well with a 200-μL sterile pipette tip. The cells were then washed with PBS to remove debris and serum-free medium was added to each well. Images were captured at 0 and 48 h after the scratch using an inverted microscope.

### Kaplan–Meier analysis

The Kaplan–Meier analysis, as well as the Log-rank test, were conducted to perform the survival analysis. Kaplan–Meier analysis is a commonly used method in survival analysis to estimate the probability of survival over time for a group of individuals. It is often used in medical research to analyze the survival rates of patients with certain conditions or diseases. The analysis is based on a survival curve, which shows the proportion of individuals who survive over time. The significance of Kaplan–Meier analysis lies in its ability to estimate survival probabilities and identify differences in survival rates between groups, which can help in the development of better treatment strategies and patient care.

### Data analysis

All data processing, statistical analysis, and plotting were conducted in R 4.0.5 software. Correlations between two continuous variables were assessed via Pearson’s correlation coefficients. The chi-squared or fisher exact test was applied to compare categorical variables, and continuous variables were compared through the Wilcoxon rank-sum test or T test (Liu et al. 2022). The *p-value* < 0.05 was established as the criterion for determining statistical significance.

## Result

### Determination of functional genomic genes in low-grade glioma

**Fig. 2:**
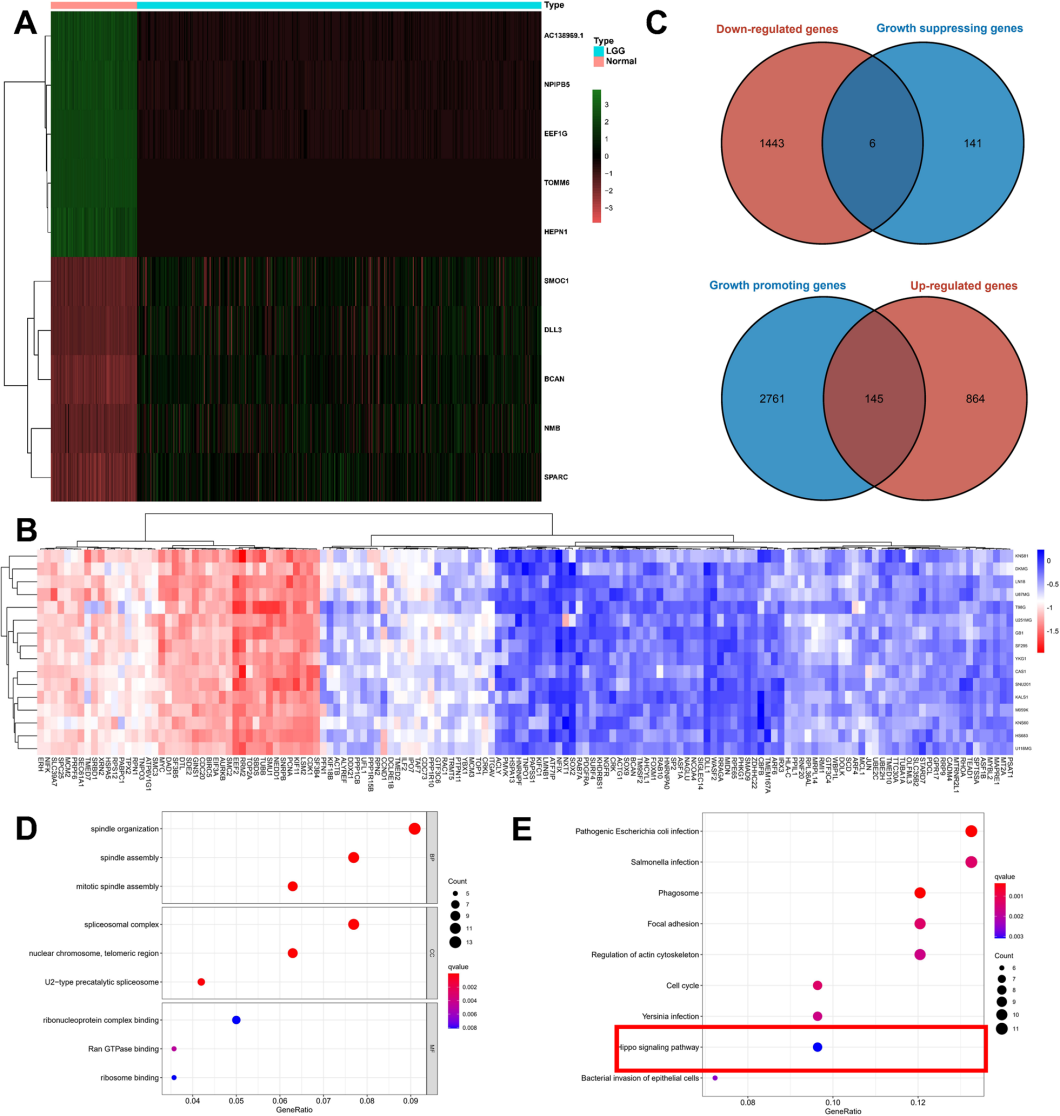
Oncogene determination with the aid of the TCGA and Depmap datasets. **A** The top ten genes with differential expression between tumor and normal samples in the TCGA dataset. **B** Oncogenes’ dependence scores in primary glioma cell lines. **C** Genes that were overlapped in both the TCGA and Depmap datasets. **D** A total of 45 genes were subjected to GO analysis. **E** A total of 45 genes were subjected to KEGG analysis

Heat map shows the Top 10 differential genes expressed in TCGA (Additional file 8, Fig. 2A). Cell lines of 16 primary gliomas were scored for dependency. Figure 2B depicts the scores assigned to oncogenes according to their dependence on other genes. The genes with dependency scores below − 0.5 in all glioma cell lines intersected with genes that were up-modulated in TCGA, and 145 genes were found (Additional file 8). Moreover, genes whose dependency scores were above 0.5 in all glioma cell lines were intersected with genes downmodulated in TCGA, yielding only six genes (Fig. 2C). The 145 genes identified were designated as the oncogenes. The three most enriched pathways in GO analysis were spindle organization, spliceosomal complex, and ribonucleoprotein complex binding (Fig. 2D). Interestingly, the findings obtained from KEGG analysis demonstrated enrichment of these oncogenes in the cell cycle, Hippo signaling pathway, and other pathways (Fig. 2E). The PPI network shows that these proteins contain 150 nodes with 794 edges between them (Additional file 2: Fig. S1A). Nodes in the context of our study represent the genes, and edges represent the interactions between them. These interactions can be physical interactions such as protein–protein interactions or functional interactions such as co-regulation. Overall, the mutation rates of all these genes were low, with only PDGFRA, SRBD1, CCDN1, CDC73, and ACLY having > 1% mutation frequency among the 145 oncogenes (Additional file 2: Fig. S1B), and no significant mutations in the other six intersecting genes (Additional file 2: Fig. S1C).

### Creation and verification of the gene signature

The “sva” package was used for background correction, normalization, and expression calculation for the genes involved in their modeling (Additional file 3: Fig. S2). With the aid of the Lasso regression model, a sum of 19 genes was filtered and used to construct risk equations. Additional file 1: Table S1 contains the coefficients derived. In the internal training dataset, a median risk score of 0.752324 was used as the threshold to classify patients into low- and high-risk groups, and the same threshold was used in the other validation datasets. In the modeling set, the patients’ OS was found to be remarkably improved in the low-risk group as opposed to that of the high-risk patients. In Additional file 4: Fig. S3A, B, the result indicates that the prognostic marker of risk score was valid (*p-value* < 0.001). The analytic results of the survival curves demonstrated that the LGG patients with a high-risk profile had a 3-year survival rate of about 56.34% with a 95% CI confidence interval of [47.18–67.3%]. LGG patients with a low-risk profile had a 3-year survival rate of about 97.2% with a 95% CI of [93.9–100%]; In the high-risk LGG patients, the 5-year survival rate was found to be about 37.58% with 95% CI [27.45–51.4%], much lower than that of the low-risk LGG patients who had a rate of about 89.3% with 95% CI confidence interval [78.86–100%]; For LGG patients with a high-risk profile, the 10-year survival rate was found to be about 12.54% with 95% CI [5–31.4%], whereas that of the low-risk LGG patients was about 65.1% with a 95% CI of [43.50–97.3%]. The AUC for 1, 3, and 5 years was 0.865, 0.910, and 0.874, respectively, according to the analysis results of the ROC curves for the different periods. Validation of the above results was performed in both the internal and external validation datasets. As anticipated, in the internal validation dataset, the findings recorded from the Kaplan–Meier curve demonstrated a short OS duration in patients belonging to the high-risk group as opposed to patients belonging to the low-risk group, with a statistically significant difference (*p-value* < 0.05). The analytical findings from the survival curves demonstrated that the high-risk LGG patients exhibited a 3 and 5-year survival of about 60.3% and 36.3%, respectively. In addition, 95% CI was [47.2–76.9%] and [20.4–64.5%], respectively. In low-risk LGG patients, the survival rates were about 89.1% and 62.6% at 3 and 5 years, respectively. 95% CI were [79.2–100%] and [44.0–89.0%], respectively. The analysis of ROC curves over 1, 3, and 5 years generated AUC values of 0.861, 0.878, and 0.690, respectively.

**Fig. 3:**
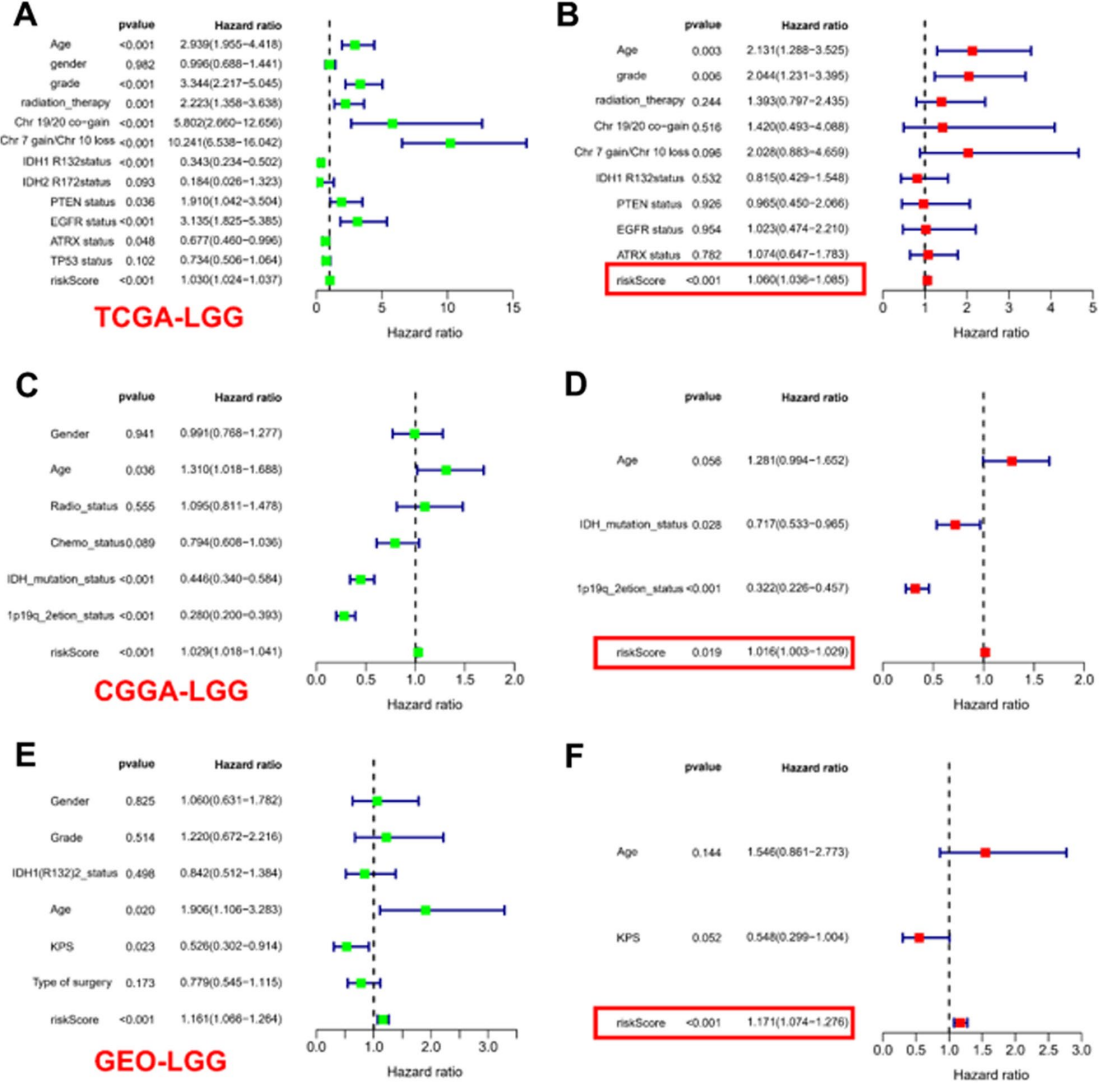
Cox regression analysis of the TCGA-LGG and validation cohorts on multivariate and univariate models. **A** Analysis of the TCGA-LGG cohort using univariate Cox regression. **B** Parameters significant in univariate Cox regression were included in multivariate Cox regression analysis in the TCGA-LGG cohort. **C** Cox regression analysis using a univariate model in the CGGA-LGG cohort. **D** Parameters significant in univariate Cox regression were included in multivariate Cox regression analysis in the CGGA-LGG cohort. **E** Analysis of the GEO-LGG cohort using univariate Cox regression. **F** Parameters significant in univariate Cox regression were included in multivariate Cox regression analysis in the GEO-LGG cohort

**Fig. 4:**
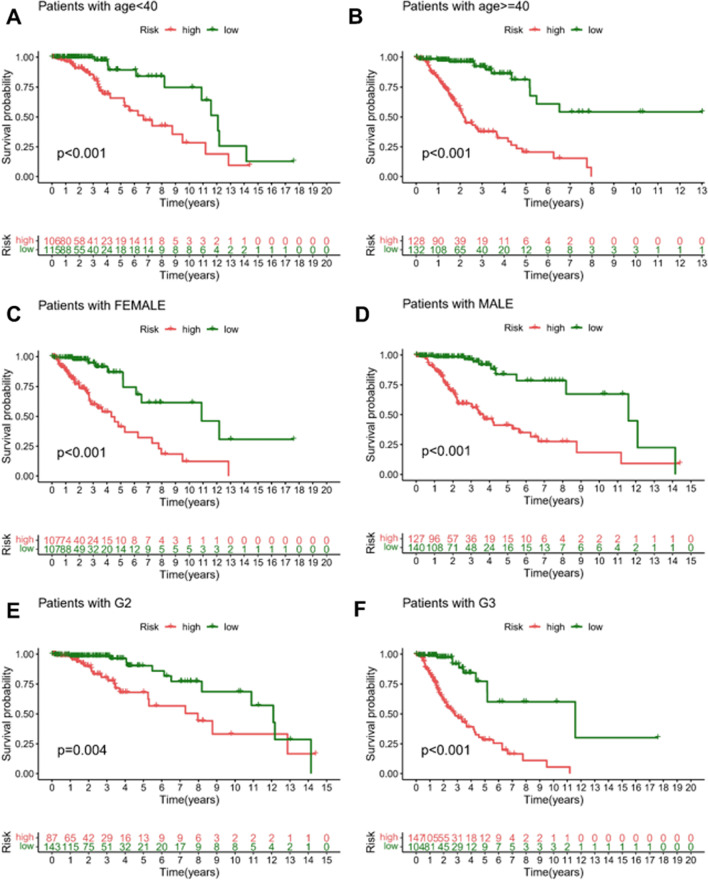
The Kaplan–Meier plot depicts the risk score for each subtype. Kaplan–Meier plot of the risk score in patients with age < 40 (**A**), patients with age ≥ 40 (**B**), patients with female (**C**), patients with male (**D**), WHO Grade 2 (**E**), and WHO Grade 3 (**F**) in the TCGA-LGG dataset

In the CGGA-LGG-1 cohort, the results obtained by the survival curves demonstrated a remarkably shortened OS duration in the high-risk patients as opposed to that of patients belonging to the low-risk group (*p-value* < 0.001). For the high-risk LGG patients, the survival rates at 3 and 5 years were about 59.4% and 46.4%, respectively. Moreover, 95% CI was [52.6–67.0%] and [39.2–54.9%], respectively. For the low-risk patients, the survival rates at 3 and 5 years were about 74.9% and 66.9%, respectively. Moreover, 95% CI were [68.1–82.4%] and [59.3–75.5%]. Overall, the AUC values recorded from the ROC curve analysis of the CGGA-LGG-1 cohort for 1, 3, and 5 years were 0.726, 0.702, and 0.703, respectively. Similarly, in the CGGA-LGG-2 cohort, high-risk patients exhibited an unfavorable OS in contrast with that of the low-risk patients (*p-value* < 0.001), with AUC values obtained from ROC curves for 1, 3, and 5 years being of 0.736, 0.767, and 0.743, respectively. Interestingly, no significant differences were observed in the GEO-LGG cohort between patients in the high- and low-risk groups in terms of OS (*p-value* = 0.051). Following the adjustment for clinical and pathological factors, Cox regression demonstrated that risk score independently served as a prognostic feature across the training set. In the entire training TCGA-LGG set (Fig. 3A, B, *p-value* < 0.001, HR: 1.060, 95% CI 1.036–1.085) and the external validation CGGA-LGG set (Fig. 3C, D, p < 0.05, HR: 1.016, 95% CI 1.003–1.029), the GEO-LGG set (Fig. 3E, F, p < 0.001, HR: 1.171, 95% CI 1.074–1.276), the risk score was shown to independently function as prognostic feature. In the TCGA-LGG full set, differences in OS stratified by clinicopathological characteristics of the universals, i.e., high- and low-risk groups, were analyzed by stratification. For the low-risk cohort, the OS remained superior in the high-risk group (Fig. 4A–F) according to subgroups classified by sex, age, tumor grade, and mutational status of ATRX, EGFR, PTEN, TP53, and IDH (Additional file 5: Fig. S4).

In addition, the predictive efficacy of independent prognostic factors was predicted in multifactorial Cox regression in the CGGA cohort and TCGA cohort. In Additional file 6: Fig. S5A, for the 3-year tROC curve, the AUC areas for age, risk score, and tumor grade were 0.487, 0.858, and 0.527 respectively for the entire TCGA-LGG dataset. For the 5-year tROC curve, the AUC areas for risk score, age, and tumor grade in the whole TCGA-LGG dataset were 0.882, 0.485, and 0.435, respectively. For the 10-year tROC curve, the AUC areas for risk score, age, and tumor grade in the whole TCGA-LGG dataset were 0.797, 0.486, and 0.465, respectively. In Additional file 6: Fig. S5B, for the 3-year tROC curve, the AUC areas for risk score, IDH mutation status, and chromosome 1p19q joint deletion in the externally validated CGGA-LGG dataset were 0.723, 0.354, and 0.371, respectively. For the 5-year tROC curve, the AUC areas for risk score, IDH mutation status, and 1p19q joint chromosome deletion in the externally validated CGGA-LGG dataset were 0.718, 0.366, and 0.340, respectively. For the 10-year tROC curve, the AUC areas for risk score, IDH mutation status, and 1p19q joint chromosome deletion in the externally validated CGGA-LGG dataset were 0.708, 0.394, and 0.318, respectively.

### DEGs between low- and high-risk subgroups

**Fig. 5:**
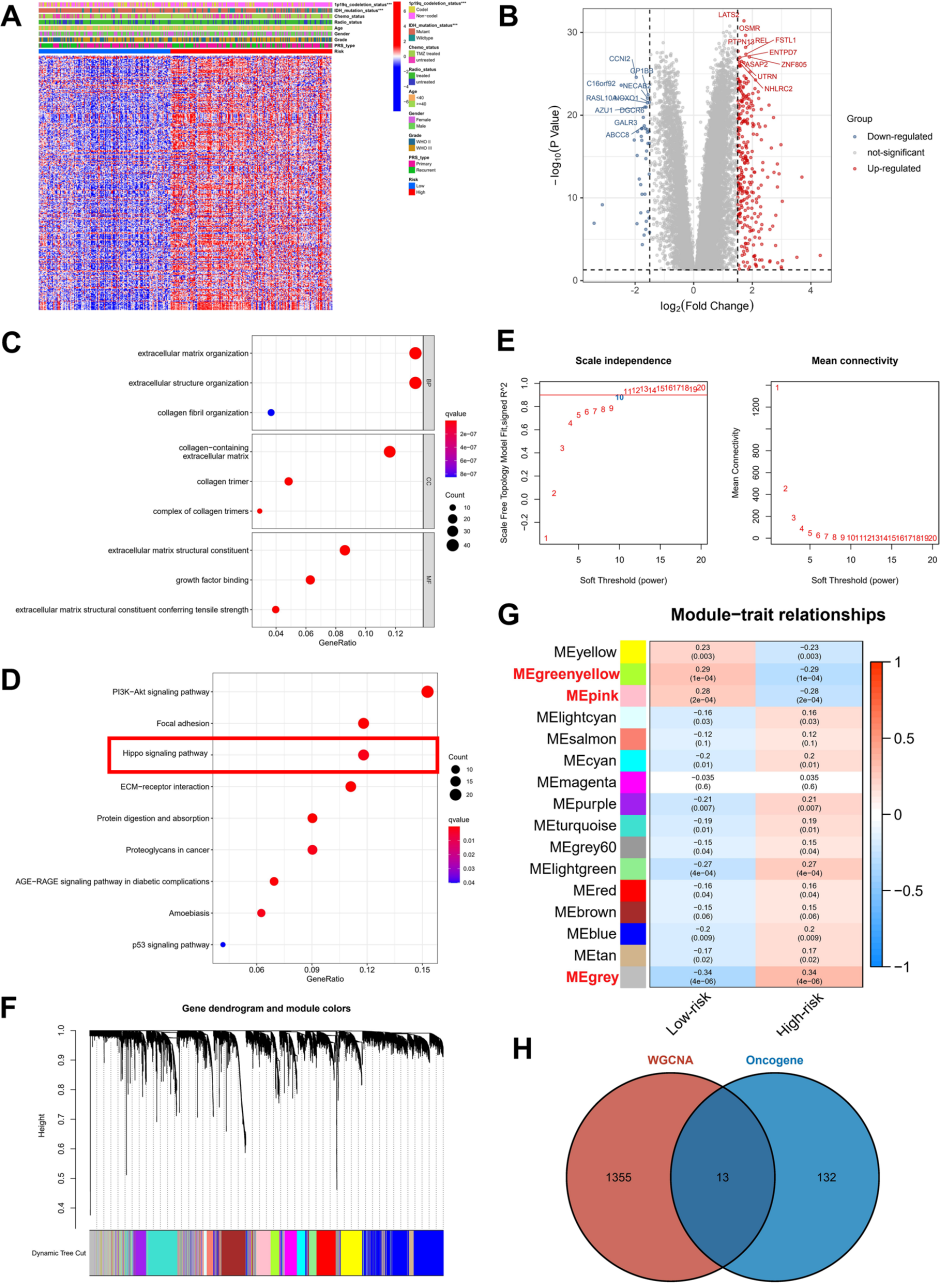
The differences in various variables between low- and high-risk patients. **A** In the CGGA-LGG-1 dataset, heatmaps show differentially expressed genes (DEGs) between high- and low-risk groups. **B** DEGs in the CGGA-LGG-1 dataset between high- and low-risk groups. **C** Up-modulated genes were analyzed using the GO algorithm. **D** Analysis of up-modulated genes using the KEGG database. **E** Scale-free fit index analysis for different soft threshold powers. **F** Modules for mRNA co-expression networks. **G** Heatmap depicting the traits relationship between low- and high-risk groups. **H** Venn diagram depicting 145 oncogenes and WGCNA modules that share overlapping genes

328 DEGs were identified in the CGGA-LGG-1 dataset by calculating DEGs between the high- and low-risk groups (Fig. 5A, B). These findings demonstrated an enrichment of the above DEGs in GO analysis for extracellular matrix structural constituent, collagen-containing extracellular matrix, and extracellular matrix organization (Fig. 5C). Moreover, they were also enriched in KEGG analysis in the p53 signaling pathway, Hippo pathway, and P13K-Akt signaling pathway (Fig. 5D). Interestingly, the Hippo pathway was shown to be a crucial mechanism in regulating cell viability both in our signature and in the DEG enrichment analysis.

### WGCNA

**Fig. 6:**
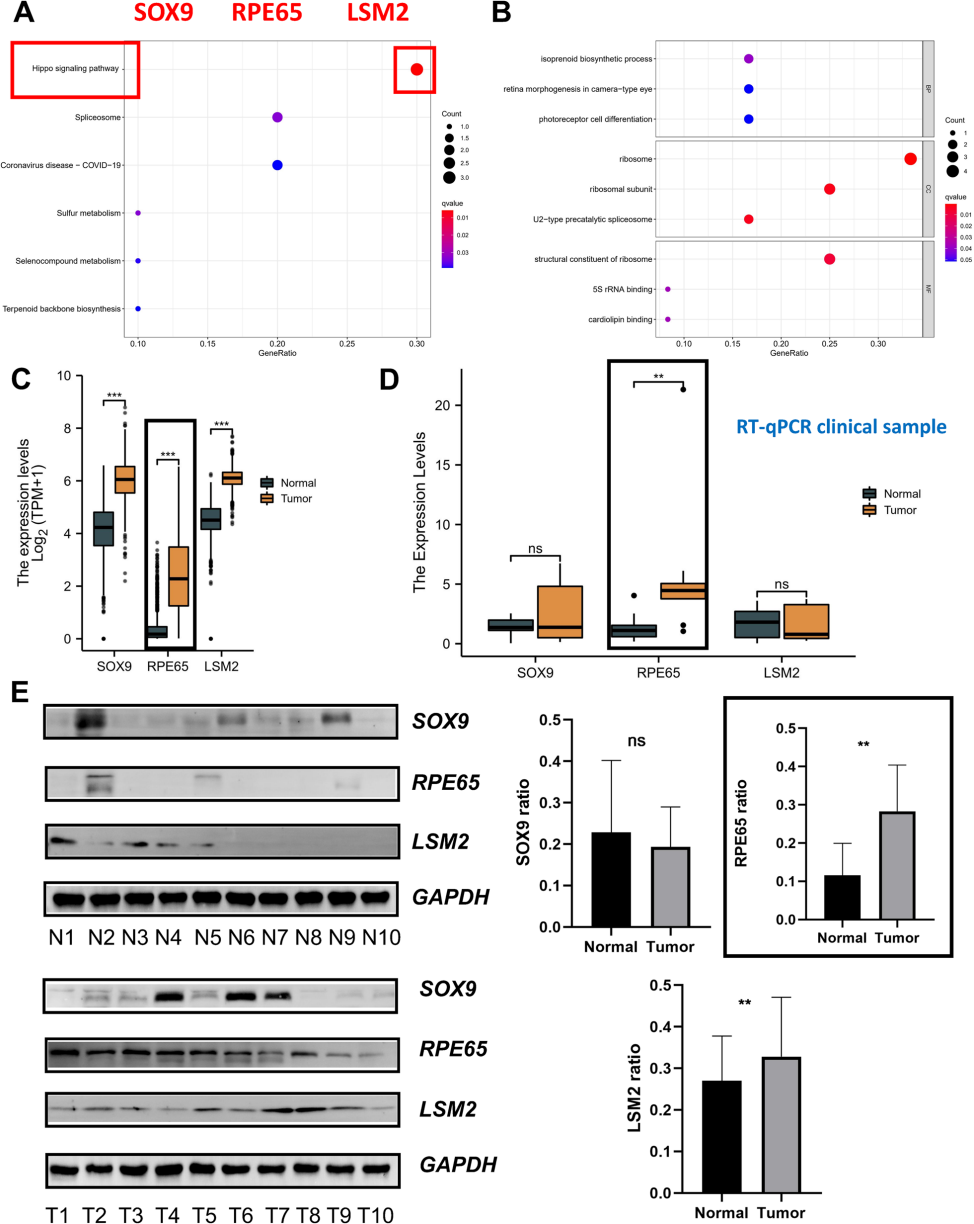
Validation of SOX9, RPE65, LSM2 using qRT-PCR and western blot in clinical samples. **A** Analysis of overlapping genes using the KEGG database. **B** GO analysis of genes that are overlapping. **C** mRNA expression of *SOX9*, *RPE65*, *LSM2* in public database. **D** The mRNA expression of *SOX9*, *RPE65*, *LSM2* in our cohort (n = 10). **E** The protein expression of *SOX9*, *RPE65*, *LSM2* in our cohort (n = 10). *P < 0.05, **P < 0.01, ***P < 0.001, ****P < 0.0001. Error bars indicate mean ± SD

The WGCNA method was adopted for the purpose of identifying hub genes in the CGGA-LGG-1 dataset. The soft threshold power was set as 10 to guarantee that the network was scale-free (Fig. 5E). With a cutoff height of 0.2, 16 clusters were clustered (Fig. 5F). It was discovered that different modules were correlated with the high-risk group (Fig. 5G), which included the green-yellow module (correlation coefficient = 0.29, *p-value* < 0.001), pink module (correlation parameter = 0.28, *p-value* < 0.001), and grey module (correlation coefficient = 0.34, *p-value* < 0.001). Genes from the three CGGA-LGG-1 datasets overlapped with 145 oncogenes, yielding a total of 13 genes (Fig. 5H). The topmost 3 enriched pathways identified by the KEGG enrichment analysis comprised the Hippo pathway, spliceosome, and Coronavirus Disease 2019 (COVID-19) (Fig. 6A). In GO analysis, the topmost 3 genes with high enrichment levels among these 13 genes were ribosome, ribosomal subunit, and structural constituent of ribosome (Fig. 6B). Among the 13 overlapping genes, three genes (SOX9, RPE65, LSM2) were enriched in the Hippo pathway.

### Validation of SOX9, RPE65, LSM2 in clinical samples

**Fig. 7:**
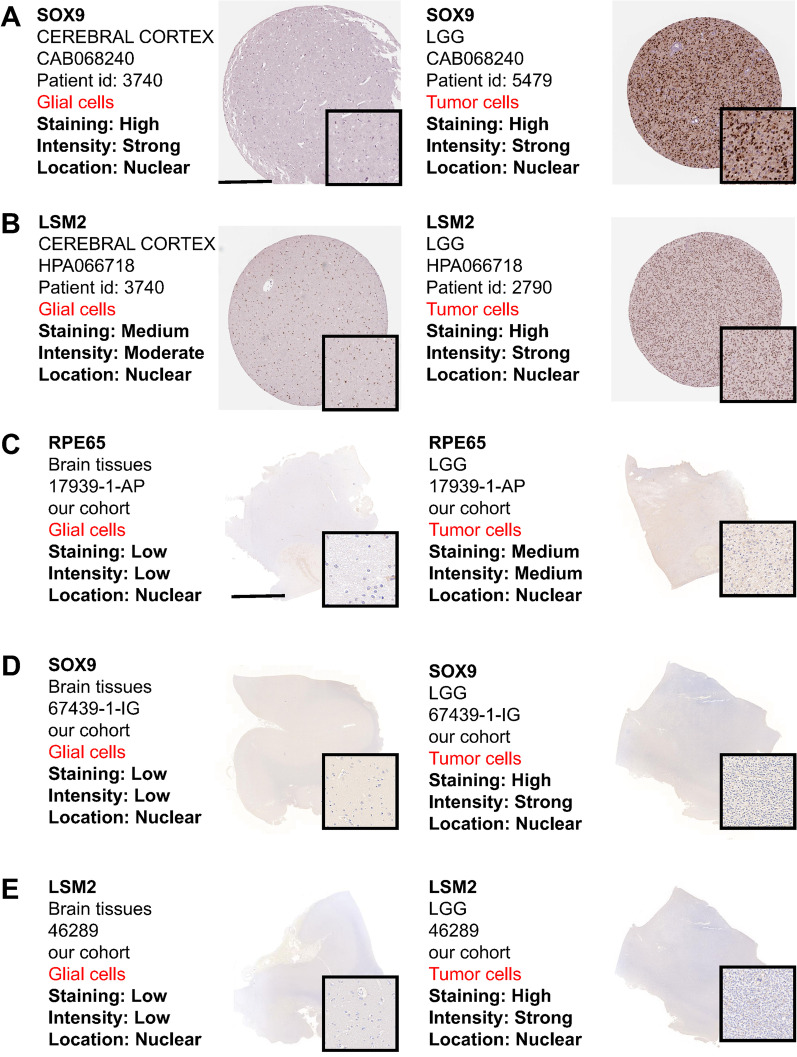
Validation of SOX9, RPE65, LSM2 using IHC. **A** Representative IHC staining images of *SOX9* in HPA database. **B** Representative IHC staining images of *LSM2* in HPA database. **C** Representative IHC staining images of *RPE65* in our cohort (n = 10). **D** Representative IHC staining images of *SOX9* in our cohort (n = 10). **E** Representative IHC staining images of *LSM2* in our cohort (n = 10). **A**, **B** Scale bars = 500 μm; **C**–**E** Scale bars = 500 μm

To verify the reliability of the hub genes (SOX9, RPE65, LSM2) obtained from the bioinformatics analysis, we explored the abnormal expression of hub genes in LGG samples combined with normal cortical samples from the GTEx database. The results showed three hub genes were highly expressed in tumor samples (Fig. 6C). Moreover, we collected 10 LGG samples and 10 normal brain tissues to detect the expression level of hub genes by qRT-PCR and western blot. In the results of qRT-PCR, the mRNA level of RPE65 were highly expressed in LGG samples, however, there was no difference in the expression of other genes in different samples (Fig. 6D). In the results of western blot, the protein level of RPE65, LSM2 were highly expressed in LGG samples (Fig. 6E). Finally, we used IHC section to further detect the expression of SOX9, RPE65, LSM2 (Fig. 7). There is no doubt that the expression of the three genes was significantly different for both IHC in the public database and in our cohort. Importantly, protein and mRNA expression of RPE65 was significantly up-regulated in tumor cells in different cohorts. Moreover, we explored the differences in RPE65 in WHO grades as well as pathological types, and the results showed that RPE65 did not differ significantly between grades (Additional file 7: Fig. S6A). Interestingly, RPE65 was lowest in oligodendroglioma compared to other pathological types (Additional file 7: Fig. S6B).

### In vitro assays

**Fig. 8:**
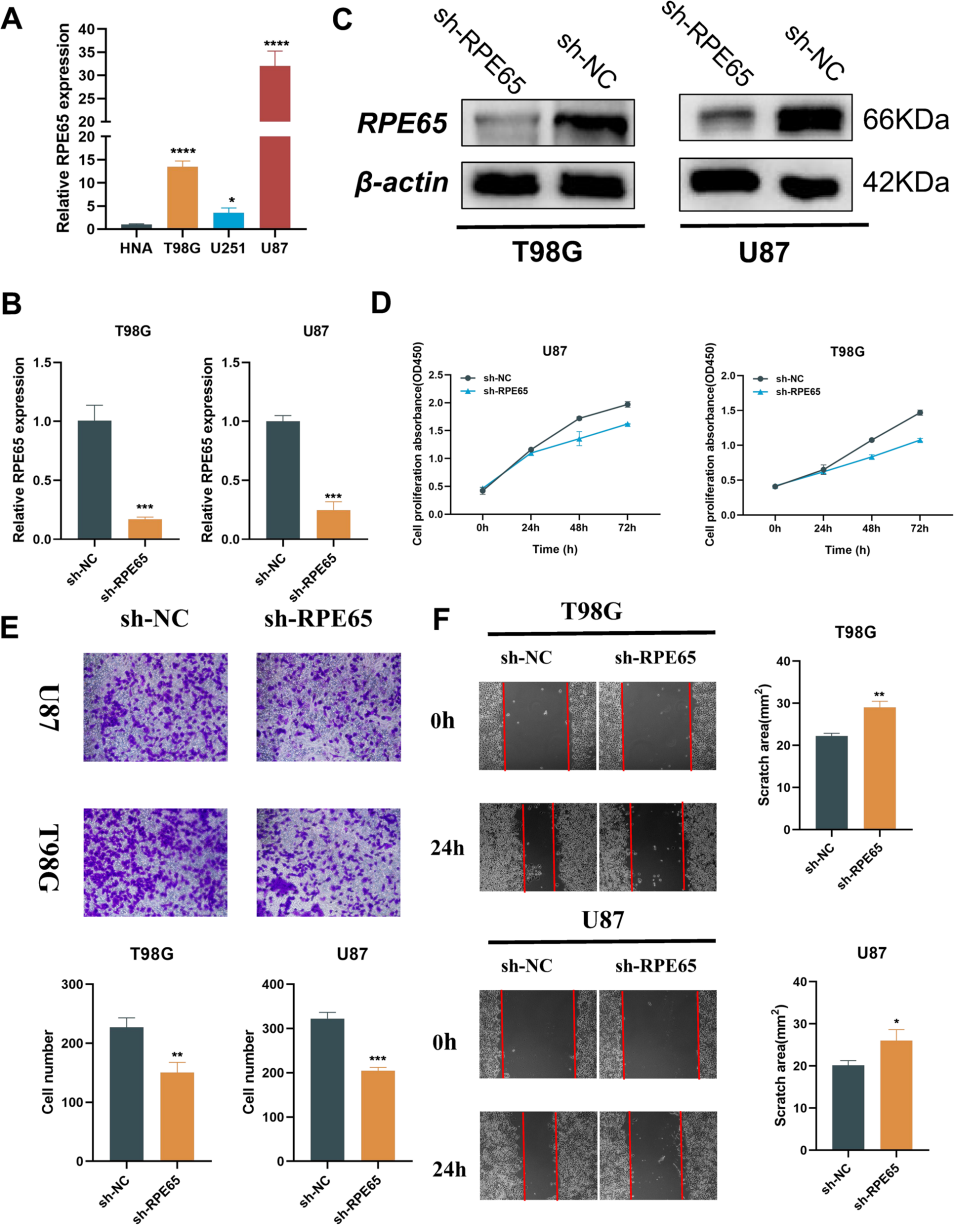
RPE65 in vitro. **A** The expression level of *RPE65* in different cell lines. qRT-PCR (**B**) and western blot (**C**) was used to analyse the expression level of *RPE65* in transfection by sh-NC or sh-RPE65 for 24 h. **D** Cell viability of U87 or T89G cells after knocking down RPE65 was determined using CCK8 assays. **E** Invasion ability of cells after knocking down RPE65 was determined using transwell assays and quantify in T98G and U87 cells with box plots. **F** Migration ability of cells after knocking down RPE65 was determined using wound healing assays and quantify in T98G and U87 cells with box plots (magnification and wound healing: ×100). *P < 0.05, **P < 0.01, ***P < 0.001, ****P < 0.0001. Error bars indicate mean ± SD

RPE65 was filtered as the candidate molecule to perform cell function assays. Real-time qPCR analysis indicated that RPE651 was significantly up-regulated in three glioma cell lines (Fig. 8A). The knock-down efficiencies of sh-RPE65 were detected using western blot and real-time qPCR, which revealed highest transfection efficiency in T98G and U87 cells (Fig. 8B, C). The CCK-8 assay showed that the viability of T98G and U87 cells was suppressed (Fig. 8D). Importantly, the invasion and migration ability of tumor cell lines was suppressed after transfection and quantify in T98G and U87 cells with box plots (Fig. 8E, F).

## Discussion

The incidence of LGG is increasing worldwide (Zhang et al. 2020). LGG patients have a relatively favorable prognosis. However, these gliomas frequently advance to high-grade gliomas, which may cause reoccurrence or death due to malignant biological characteristics, including aggressive proliferation and radiotherapy resistance. Recent studies reported that LGG tumor markers are highly predictive of prognosis and successful treatment (Smith and Schwartz 1984). These molecular markers are EGFR mutation status, PTEN mutation status, MGMT promoter methylation, TP53 mutation status, 1p/19q chromosome deletion, ATRX mutation status, and IDH1 mutation status (Roszkowski et al. 2016). The cause of LGG is characterized by various alterations in oncogenes. Crispr-cas9 screening, as a foundational technique, is an excellent approach to identify synthetic lethal genes in a systematic manner (Hewitt et al. 2021). The present research combined the findings of DepMap’s low-grade glioma CRISPR-cas9 screen with the TCGA dataset, yielding a total of 145 oncogenes that are abundantly expressed and lethal to LGG. According to the results of the enrichment analysis, the Hippo signaling pathway was shown to be significantly enriched in these genes. In addition, a gene signature was constructed from the 145 genes, which might be used to classify patients into 2 groups, namely the high- and low-risk groups. Moreover, the Hippo signaling pathway was also significant when analyzing DEGs between high- and low-risk groups and genes. The intersection between the 145 oncogenes, WGCNA analysis, and DEGs also highlighted the Hippo pathway, indicating its importance for the viability of LGG.

The changes in gene expression profile based on CRISPR/Cas9 system from DepMap database would enable the identification of hub genes, to decipher the molecular mechanism of LGG. In our study, we found three Hippo signaling pathway-related genes are mainly involved in the functional regulation. SOX9 (SRY-Box Transcriptional Factor 9) is a Protein Coding gene that performs an integral role in encoding a protein that distinguishes the CCTTGAG sequence in addition to other members of the HMG box class of DNA binding proteins (Wang et al. 2007). SOX9 plays a role in chondrocyte differentiation and is involved in the protein kinase activity and DNA-binding transcriptional factor (Ye et al. 2020). It has been demonstrated that SOX9 enhances the epithelial-mesenchymal transitions in gastric cancer cells via the mechanism of activating the Hippo-YAP signaling pathways (Zhou et al. 2019). In addition, the deletion of the YAP1 or SOX9 genes through CRISPR/Cas9 inhibited PPARδ-induced oncogenic activity. PPARδ collaborates with the hippo coactivator YAP1 to elevate the expression level of SOX9 and the progression of gastric cancer (Song et al. 2020). SOX9 expression is correlated with an unfavorable prognosis and TMZ resistance in GBM patients (Xu et al. 2018). Despite the fact that SOX2-SOX9 serves as an oncogenic axis that modulates the characteristics of stem cells and the resistance to chemotherapy. On the other hand, rapamycin attenuated the production of SOX protein, and it was found that combining rapamycin and temozolomide suppressed the progression of glioma in cells that expressed high levels of SOX2/SOX9 (Garros-Regulez et al. 2016). In patients with glioma, a higher level of SOX9 expression is correlated with an unfavorable prognosis (Wang et al. 2012). The results show that SOX9 is essential to the survival of LGG. Further research discovered that Hippo coactivator YAP1 stimulates SOX9 expression in esophageal cancer cells, resulting in these cancer cells exhibiting stem cell-like characteristics (Song et al. 2014). SOX9 trans-activated long non-coding RNA NEAT1, which stimulates self-regeneration of hepatocellular carcinoma stem cells via the PKA/Hippo pathway (Cheng et al. 2021). RPE65 (retinoic acid isomerase RPE65) is a protein-coding gene, and activity of RPE65 could be essential to the elimination of all-trans retinal, which are substrates for the synthesis of retinoic acid from skin cells (Amann et al. 2012). In addition, deletion of retinal pigment epithelial extracellular signal-regulated kinase 1/2 leads to reduced RPE65 and retinal degeneration (Pyakurel et al. 2017). LSM2 (LSM2 homolog, U6 small nuclear RNA, and mRNA degradation-related) belongs to the LSM family of encoded RNA-binding proteins. It has been shown to perform a function in pre-mRNA splicing as a constituent of the U4/U6–U5 tri-snRNP complex involved in the assembly of the spliceosome and as a constituent of the catalytic pre-snRNP complex (spliceosome B complex) (Bertram et al. 2017). A particular binding site for the heptameric LSM2-8 complex is the 3′-terminal U-bundle of snRNA U6 (Achsel et al. 1999). A previous study shows that LSM2-8 and XRN-2 contribute to the silencing of H3K27me3 marker genes by targeting RNA decay (Mattout et al. 2020). By facilitating the decay of these RNA transcripts, LSM2-8 and XRN-2 are able to prevent the expression of the genes they derive from, ultimately leading to their silencing. Overall, the findings of this previous study suggest that RNA decay is an important mechanism for regulating gene expression, and that LSM2-8 and XRN-2 are key players in this process. The findings of the CRISPR-cas9 screen indicate that the suppression of these genes resulted in cell death in all kinds of LGG. The antagonism of these genes could be a feasible method for the treatment of LGG. The overexpression of these genes in LGG may be a promising target for drug development in the future.

The limitations of our study also need to be discussed. Firstly, although some clinical samples and vitro assays have been conducted to validate, there is no evidence based on mass of clinical data to verify our prediction results. Our follow-up studies are focusing on correlation between REP65 and Hippo pathway. Despite previous research creating predictive signatures based primarily on gene expression, gene expression was integrated with functional genomic screening in the present research. Irrespective of their tumor grade, the genetic profile can distinguish between high and low-risk individuals. Therefore, suitable targeted medications may be used for the purpose of enhancing the prognosis of patients with a high risk. The prediction performance of the proposed signature is superior to classical clinicopathological parameters, such as IDH1 mutation status, tumor grade, or 1p19q staining combined with ablation. However, molecular subtyping and tumor grading are widely used in clinical practice. Apart from age and tumor grade, this signature is the only independent prognostic variable in multivariable logistic regression.

## Conclusion

In summary, this study systematically investigates genes susceptible to cell viability, and the Hippo signaling pathway is crucial to this process. The gene signature proposed in this study was combined with a functional genomic screening that predicted the prognosis of patients with LGG more accurately in contrast with conventional clinicopathological markers. These genes associated with the Hippo signaling pathway can be targeted for the treatment of LGG.

## Supplementary Information

**Additional file 1: Table S1.** The coefficients of each risk gene.

**Additional file 2: Figure S1.** Landscape of Mutation. (A) The PPI network consisting of 145 genes. (B) Rate of gene mutation in the TCGA-LGG dataset regarding 145 genes (Only PDGFRA, SRBD1, CCDN1, CDC73, and ACLY have > 1% mutation frequency). (C) No significant mutations in the six overlapped genes.

**Additional file 3: Figure S2.** Processing of data batch effect. A-B. Patients with LGG were shown to be distributed in two separate distribution clusters, according to the results of principal component analysis (PCA).

**Additional file 4: Figure S3.** Area under curve (AUC) curve and Kaplan–Meier plot derived from gene signature data for discovery and validation cohorts. (A) AUC of tROC curve for survival over 1, 3, and 5 years in TCGA-LGG-1 internal training cohort, TCGA-LGG-2 internal validation set, CGGA-LGG-1 external validation cohort, CGGA-LGG-2 external validation cohort, and GEO-LGG external validation cohort. (B) Kaplan–Meier plot of TCGA-LGG-1 internal training cohort, TCGA-LGG-2 internal validation set, CGGA-LGG-1 external validation cohort, CGGA-LGG-2 external validation cohort, and GEO-LGG external validation cohort.

**Additional file 5: Figure S4.** Kaplan–Meier curve for patients with different clinicopathologic features. (A, B) Analysis of survival time using the Kaplan–Meier curve for patients with ATRX wild type and ATRX mutant in the high- and low-risk groups. (C, D) Patients with EGFR wild-type and EGFR mutation groups were classified into high- and low-risk groups, and their overall survival (OS) was analyzed using a Kaplan–Meier curve. (E–F) Patients with PTEN wild-type and PTEN mutant groups were analyzed using the Kaplan–Meier method for the purpose of determining their OS. (G, H) Patients with TP53 wild-type and TP53 mutant groups were classified into high- and low-risk groups, and their OS was analyzed using a Kaplan–Meier curve. (I) Kaplan–Meier curve study of OS in patients with IDH2 R132 wild-type in the high- and low-risk groups. (J, K) Patients with IDH2 R172 wild-type and IDH2 R172 mutant subgroups were divided into high- and low-risk groups, and their OS was analyzed using a Kaplan–Meier curve.

**Additional file 6: Figure S5.** Clinicopathologic characteristics and the risk score were evaluated according to the area under the curve (AUC) of the tROC curves. (A) tROC curves showing 3-year, 5-year and 10-year survival in the TCGA-LGG dataset for clinical and pathological characteristics and risk score. (B) tROC curves showing 3-year, 5-year and 10-year survival in the CGGA-LGG dataset for clinical and pathological characteristics and risk score.

**Additional file 7: Figure S6.** The REP65 expression in different clinicopathologic characteristics. (A) The REP65 expression in different WHO grade. (B) The REP65 expression in different histological type. *P < 0.05, **P < 0.01, ***P < 0.001, ****P < 0.0001. Error bars indicate mean ± SD

**Additional file 8.** A list of DEGs, growth-suppressing-genes and growth-promoting-genes.

## Data Availability

The original contributions presented in the study are included in the article/Additional files, further inquiries can be directed to the corresponding author.

## Abbreviations

DEGs: Differentially expressed genes
LGG: Low-grade gliomas
GTEx: Genotype-Tissue Expression
TCGA: The Cancer Genome Atlas
LASSO: The least absolute shrinkage and selection operator
WGCNA: Weighted correlation network analysis
sgRNA: Single-stranded guide RNA
DepMap: Cancer Dependency Map
OS: Overall survival
PPI: Protein–protein interaction
GO: Gene ontology
TOM: Topological overlap matrix
