# Supplementary material for: CRISPR-cas9 screening identified lethal genes enriched in Hippo kinase pathway and of predictive significance in primary low-grade glioma
Source: Mol Med. 2023 May 14;29:64. doi: 10.1186/s10020-023-00652-3 (PMC10183247; doi:10.1186/s10020-023-00652-3)
Supplement: Supplementary file 1 — Additional file 1: Table S1. The coefficients of each risk gene. [file 10020_2023_652_MOESM1_ESM.docx]

| **Genes** | **Coefficients** |
| --- | --- |
| TAF7 | -0.954065183 |
| GTF3C4 | 0.308263979 |
| DAD1 | -0.411171775 |
| MRPL14 | -0.62362364 |
| XRN2 | 0.25671856 |
| TNPO1 | 0.614607849 |
| PPP1R10 | -0.648589932 |
| PPP1CB | 0.373522965 |
| TRMT5 | -0.319279925 |
| ITGAV | 0.029348933 |
| CBFB | 0.635634851 |
| ACTG1 | 0.110047584 |
| ATF7IP | -0.134629017 |
| NFIX | -0.395783471 |
| SMAD9 | -0.202889097 |
| PPP1R15B | 0.386090833 |
| SOX9 | 0.472561879 |
| TOP2A | -0.052152527 |
| BIRC5 | 0.504019854 |
