# Supplementary figures and images for: CRISPR-cas9 screening identified lethal genes enriched in Hippo kinase pathway and of predictive significance in primary low-grade glioma
Source: Mol Med. 2023 May 14;29:64. doi: 10.1186/s10020-023-00652-3 (PMC10183247; doi:10.1186/s10020-023-00652-3)

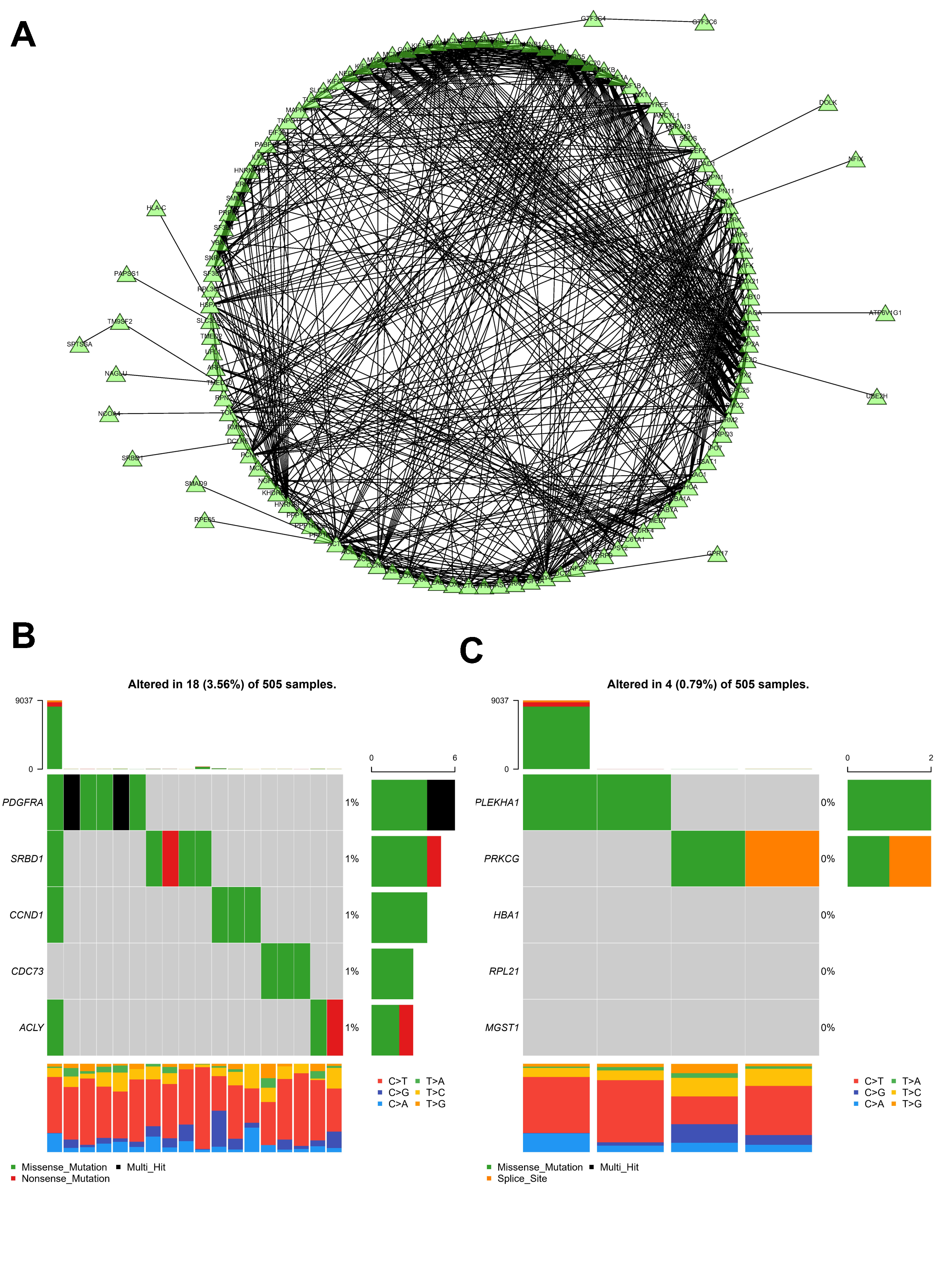

Supplement: Supplementary file 2 — Additional file 2: Figure S1. Landscape of Mutation. (A) The PPI network consisting of 145 genes. (B) Rate of gene mutation in the TCGA-LGG dataset regarding 145 genes (Only PDGFRA, SRBD1, CCDN1, CDC73, and ACLY have > 1% mutation frequency). (C) No significant mutations in the six overlapped genes. [file 10020_2023_652_MOESM2_ESM.tif]

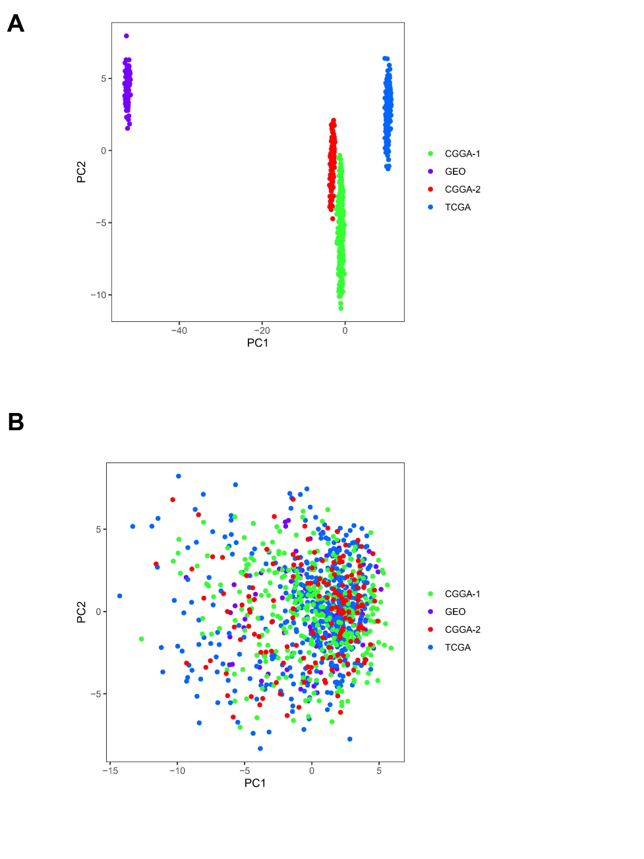

Supplement: Supplementary file 3 — Additional file 3: Figure S2. Processing of data batch effect. A-B. Patients with LGG were shown to be distributed in two separate distribution clusters, according to the results of principal component analysis (PCA). [file 10020_2023_652_MOESM3_ESM.png]

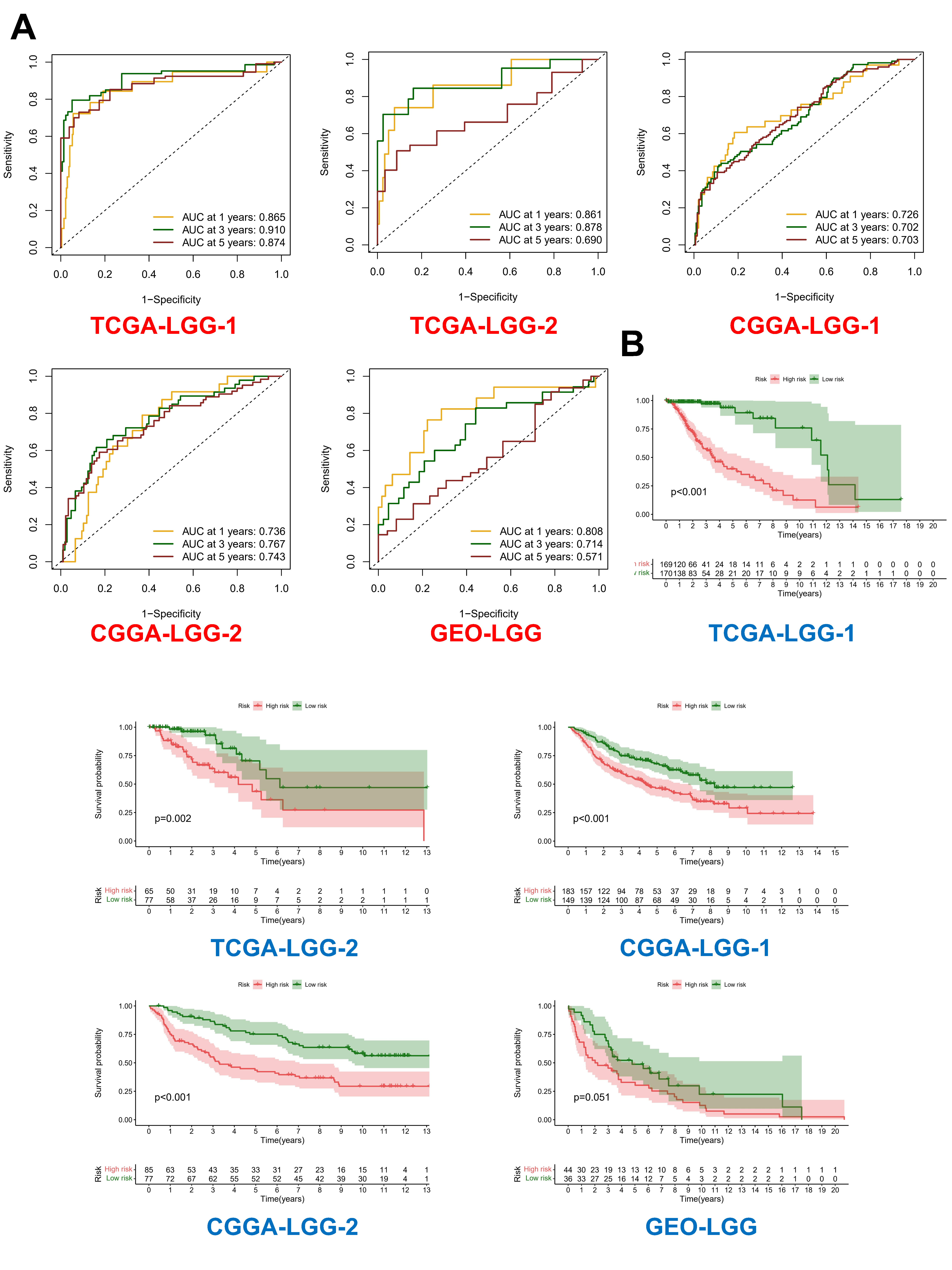

Supplement: Supplementary file 4 — Additional file 4: Figure S3. Area under curve (AUC) curve and Kaplan–Meier plot derived from gene signature data for discovery and validation cohorts. (A) AUC of tROC curve for survival over 1, 3, and 5 years in TCGA-LGG-1 internal training cohort, TCGA-LGG-2 internal validation set, CGGA-LGG-1 external validation cohort, CGGA-LGG-2 external validation cohort, and GEO-LGG external validation cohort. (B) Kaplan–Meier plot of TCGA-LGG-1 internal training cohort, TCGA-LGG-2 internal validation set, CGGA-LGG-1 external validation cohort, CGGA-LGG-2 external validation cohort, and GEO-LGG external validation cohort. [file 10020_2023_652_MOESM4_ESM.tif]

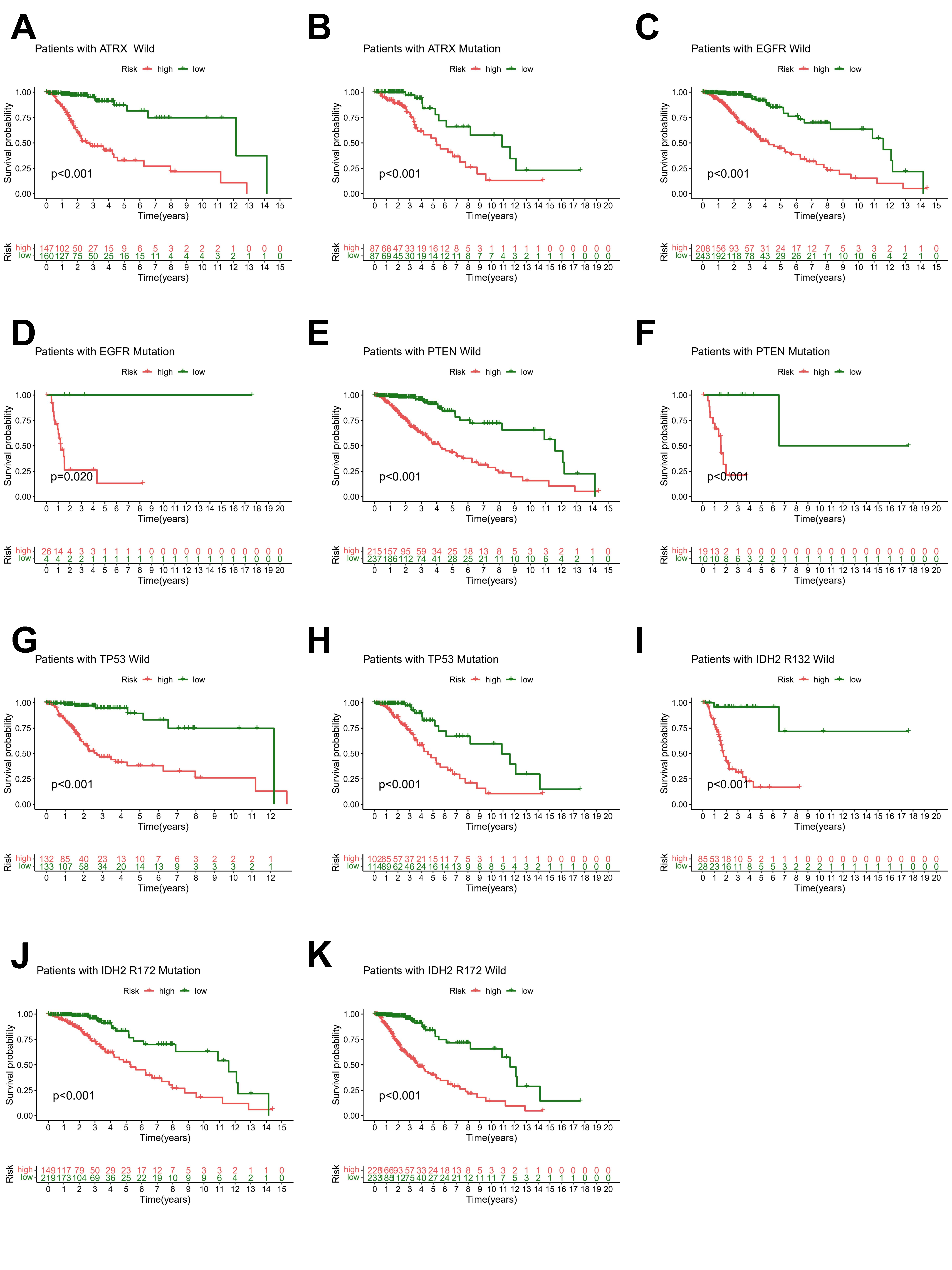

Supplement: Supplementary file 5 — Additional file 5: Figure S4. Kaplan–Meier curve for patients with different clinicopathologic features. (A, B) Analysis of survival time using the Kaplan–Meier curve for patients with ATRX wild type and ATRX mutant in the high- and low-risk groups. (C, D) Patients with EGFR wild-type and EGFR mutation groups were classified into high- and low-risk groups, and their overall survival (OS) was analyzed using a Kaplan–Meier curve. (E–F) Patients with PTEN wild-type and PTEN mutant groups were analyzed using the Kaplan–Meier method for the purpose of determining their OS. (G, H) Patients with TP53 wild-type and TP53 mutant groups were classified into high- and low-risk groups, and their OS was analyzed using a Kaplan–Meier curve. (I) Kaplan–Meier curve study of OS in patients with IDH2 R132 wild-type in the high- and low-risk groups. (J, K) Patients with IDH2 R172 wild-type and IDH2 R172 mutant subgroups were divided into high- and low-risk groups, and their OS was analyzed using a Kaplan–Meier curve. [file 10020_2023_652_MOESM5_ESM.tif]

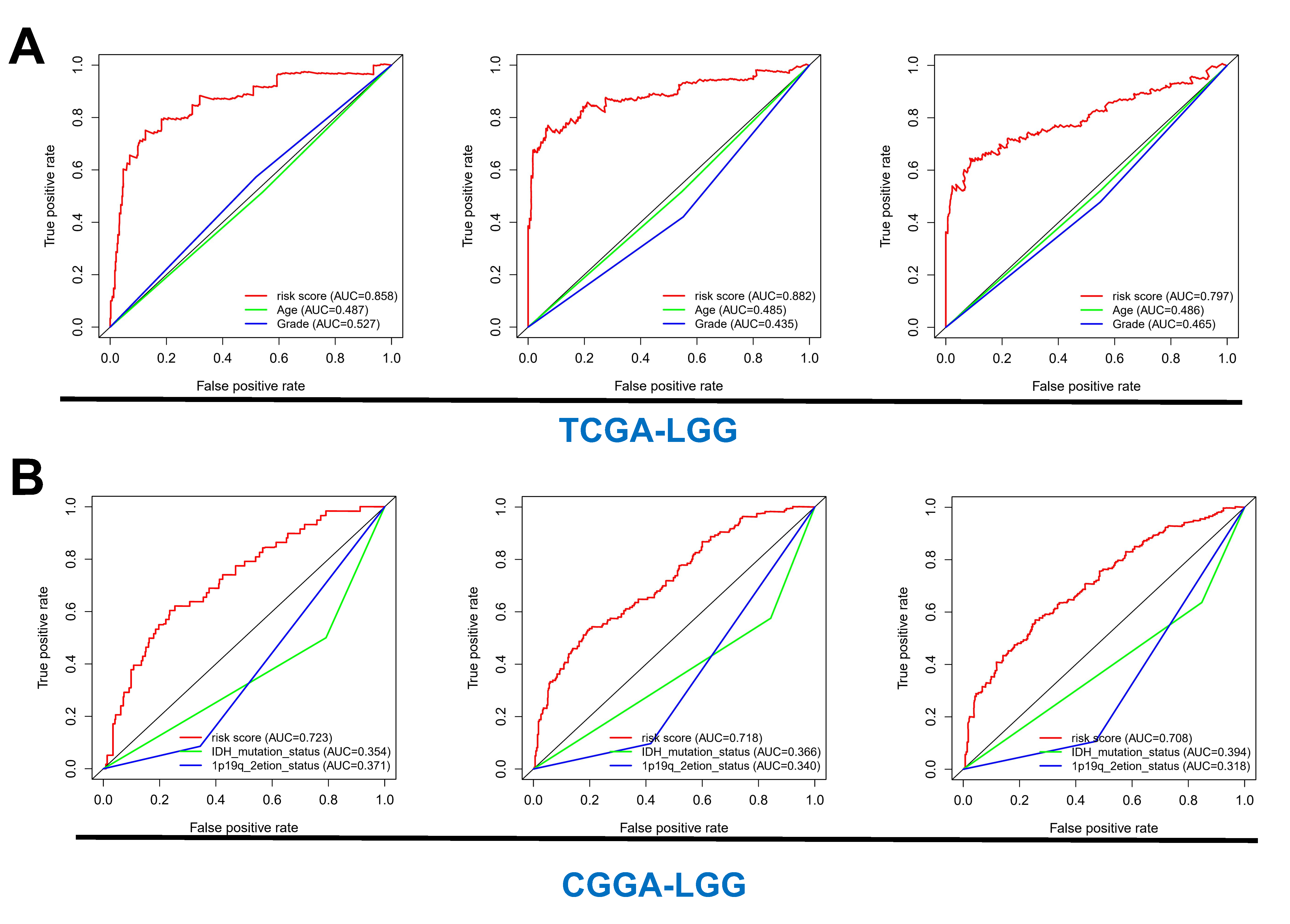

Supplement: Supplementary file 6 — Additional file 6: Figure S5. Clinicopathologic characteristics and the risk score were evaluated according to the area under the curve (AUC) of the tROC curves. (A) tROC curves showing 3-year, 5-year and 10-year survival in the TCGA-LGG dataset for clinical and pathological characteristics and risk score. (B) tROC curves showing 3-year, 5-year and 10-year survival in the CGGA-LGG dataset for clinical and pathological characteristics and risk score. [file 10020_2023_652_MOESM6_ESM.tif]

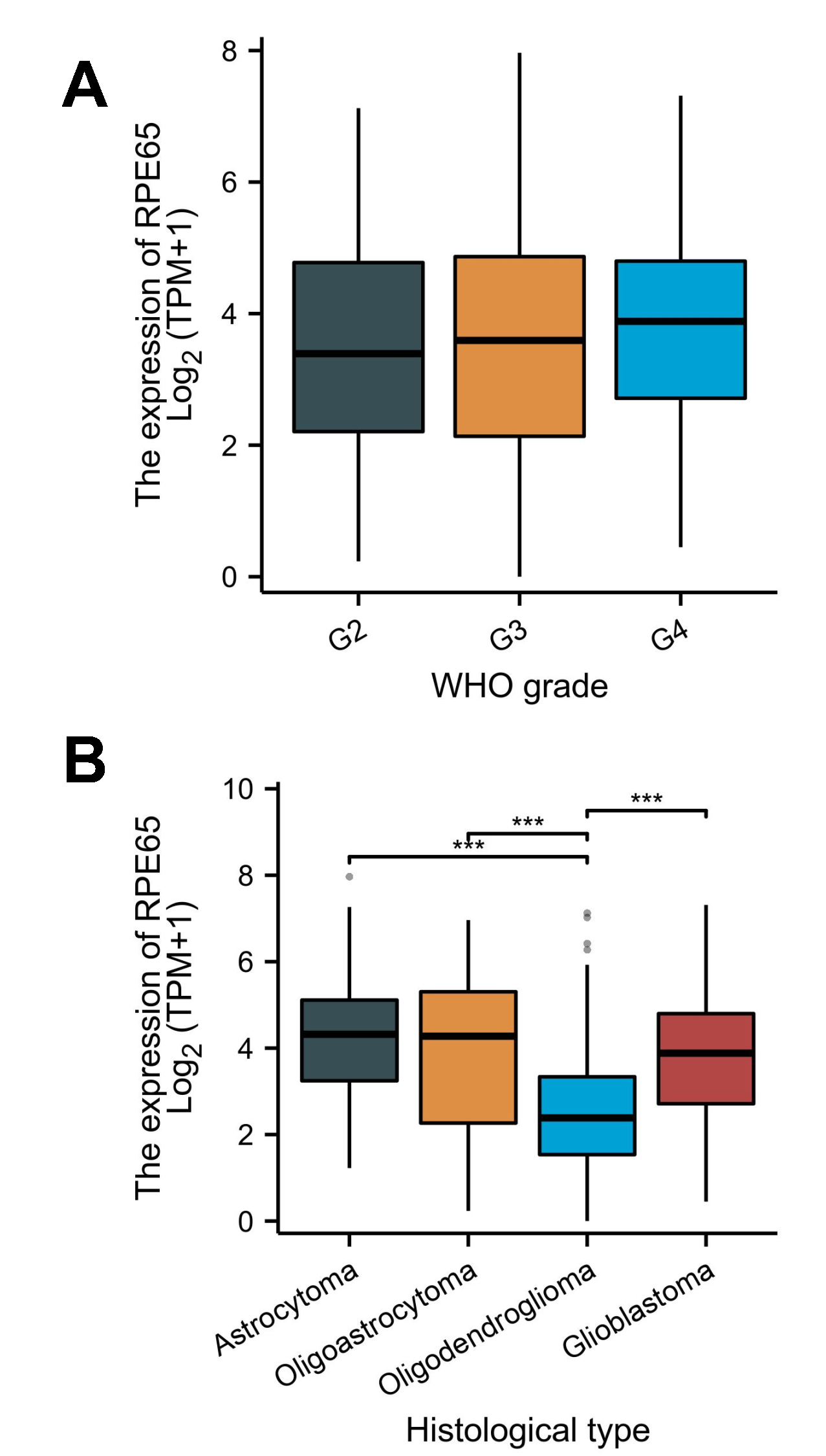

Supplement: Supplementary file 7 — Additional file 7: Figure S6. The REP65 expression in different clinicopathologic characteristics. (A) The REP65 expression in different WHO grade. (B) The REP65 expression in different histological type. *P < 0.05, **P < 0.01, ***P < 0.001, ****P < 0.0001. Error bars indicate mean ± SD [file 10020_2023_652_MOESM7_ESM.tif]
